# Supplementary material for: A thirty-year trend of increasing clinical orientation at the National Institutes of Health
Source: bioRxiv. 2025 Dec 19:2025.12.16.694423. Preprint. [Version 1] doi: 10.64898/2025.12.16.694423 (PMC12724543; doi:10.64898/2025.12.16.694423)
Supplement: Supplement 1 [file media-1.pdf]

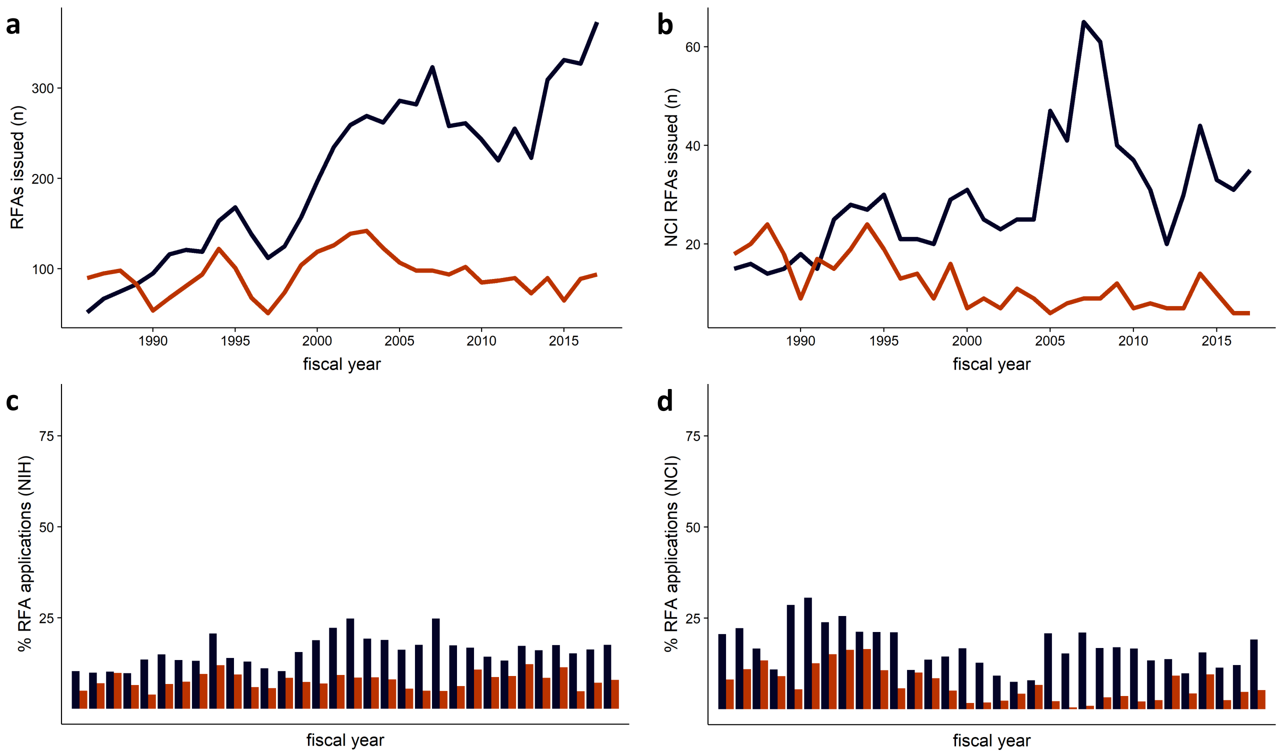

Figure S1. Minimal effect of funding opportunity announcements (FOAs) with set-aside dollars on receipt of applications. (**a, b**) Number of FOAs with set-aside dollars issued each year by NIH (left) or NCI (right) through either an R01 (red) or non-R01 (navy blue) mechanism. (**c, d**) Applications to FOAs with set-aside dollars as a percentage of all R01 (red) or non-R01 (navy blue) applications received by NIH (left) or NCI (right).

Figure S1

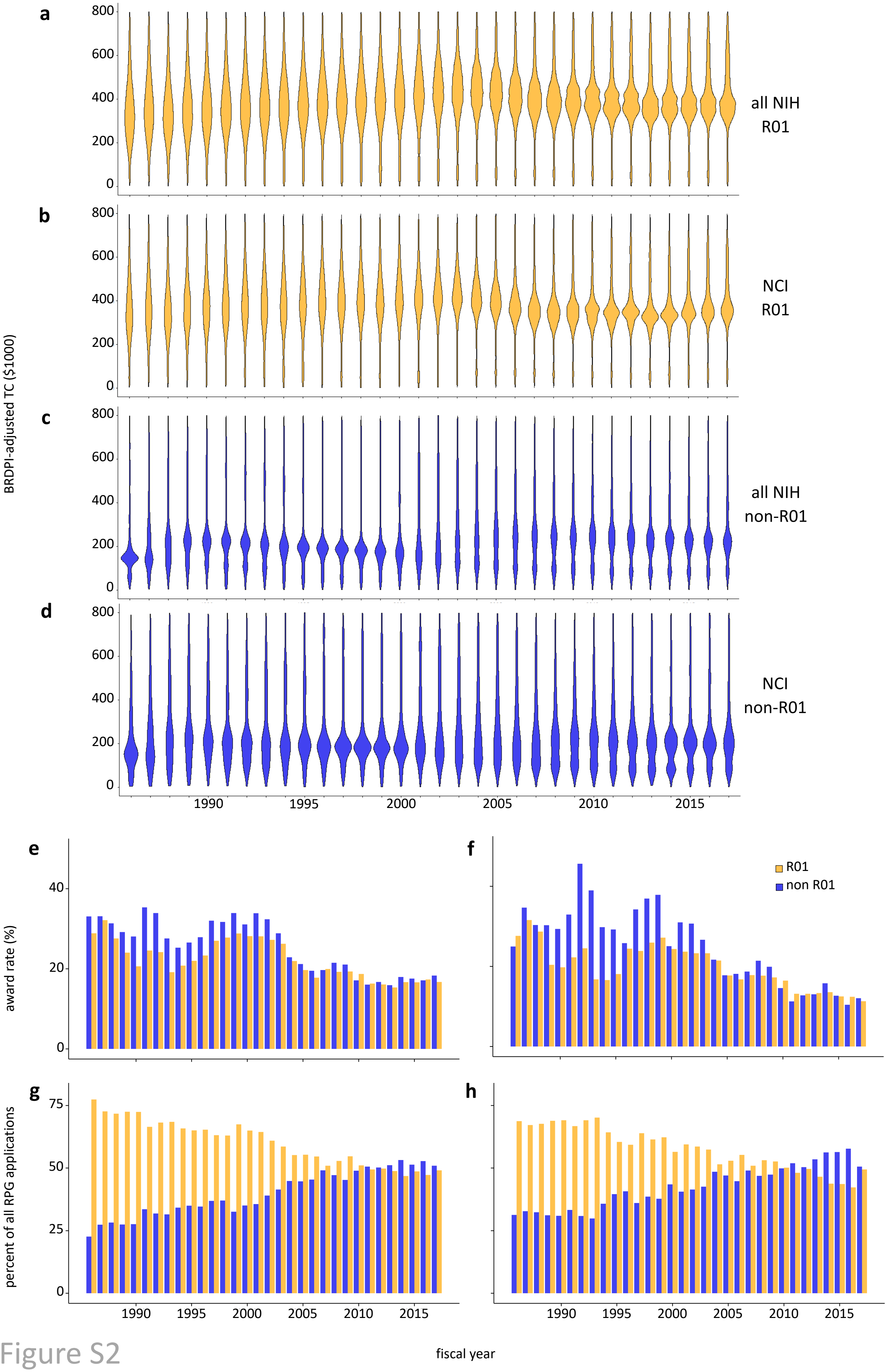
a
b
c
d
800
600
400
200
0
all NIH
R01
NCI
R01
all NIH
non-R01
NCI
non-R01
BRDPI-adjusted TC ($1000)
1990
1995
2000
2005
2010
2015
e
f
g
h
40
20
award rate (%)
R01
non R01
75
50
25
percent of all RPG applications
fiscal year

Figure S2

Figure S2. Proliferation and expansion of non-R01 mechanisms is sufficient to explain the declining number of R01 awards. Data on award size, award rate, and proportion of applications received for the NIH and the NCI between fiscal years 1986 and 2017; R01s are shown in orange and all other non-R01 RPGs in blue. Distribution of award size for the entire NIH and for the NCI is shown as total costs (TC) in thousands of dollars for (**a,b**) R01 awards and (**c,d**) all other non-R01 research project grants (RPGs), adjusted for inflation to 2017 dollars using the Biomedical Research and Development Price Index (BRDPI). Across all of NIH, the inflation-adjusted mean budget size has remained unchanged. At NCI, R01 budgets have not detectably changed, and non-R01 budgets have declined approximately $6000 each year (see Supplemental Materials for details). For (**e**) all of NIH and (**f**) the NCI, award rates over the same time frame for R01 (orange) and non-R01 (blue) applications. Also, for (**g**) NIH and (**h**) NCI over the same time frame, R01 (orange) and non-R01 (blue) applications as a proportion of all grant applications.

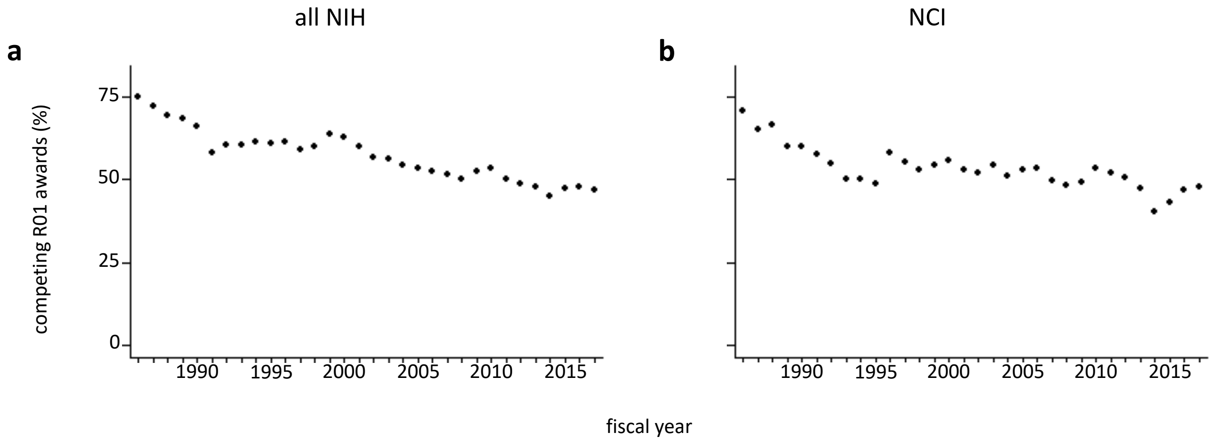

Figure S3. R01s as a relative fraction of the NIH portfolio. For (**a**) NIH and (**b**) NCI, the percentage of competing T1 and T2 R01 awards as a fraction of all research grant awards, in fiscal years 1986 to 2017.

Figure S3

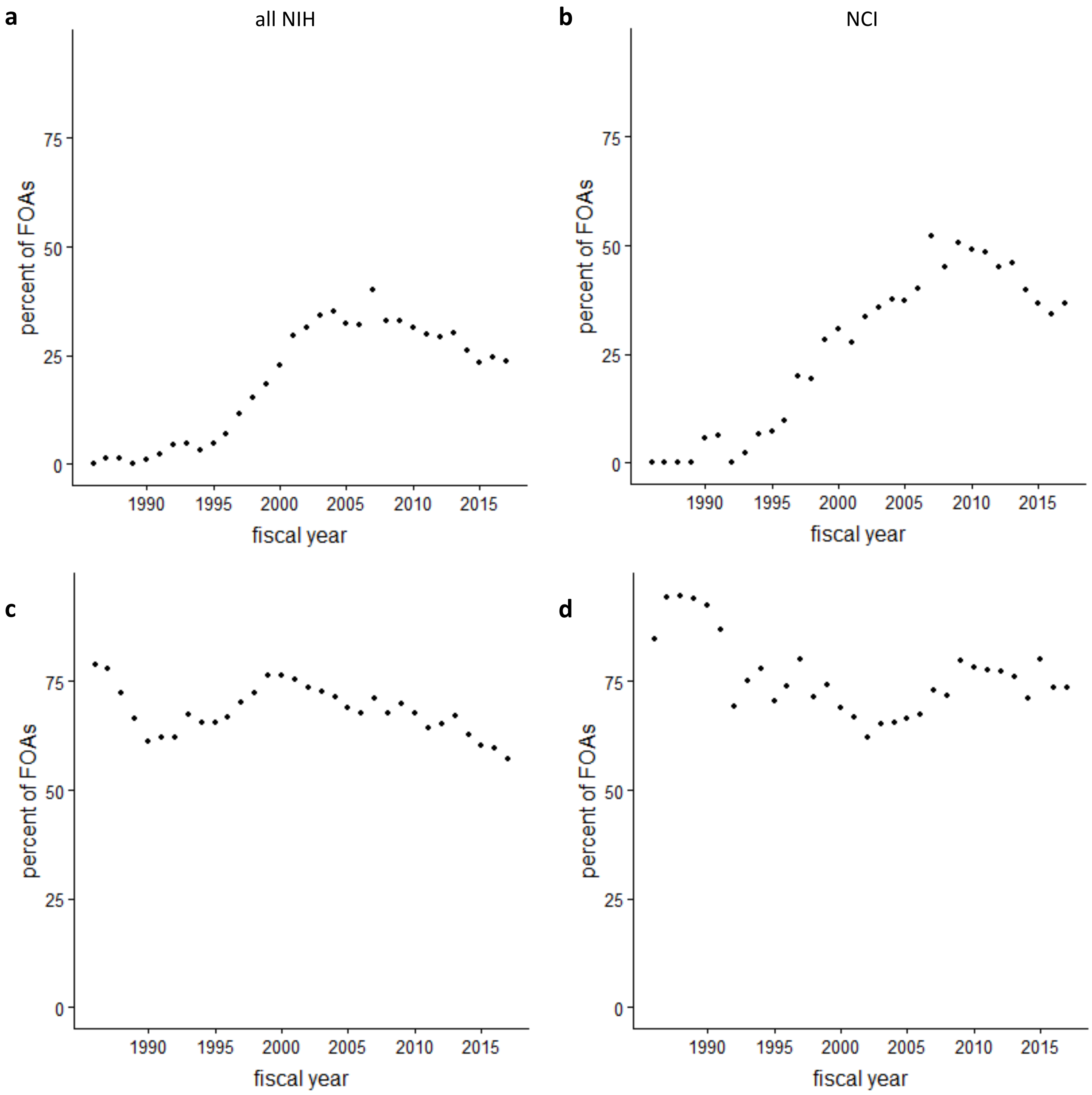

Figure S4. A small number of mechanisms account for a large percentage of funding opportunity announcements (FOAs) issued annually by NIH and NCI. (**a, b**) Percent of FOAs issued annually by NIH (left) and NCI (right) through the R21 mechanism. (**c, d**) Percent of FOAs issued annually by NIH (left) and NCI (right) through the R01, R21, R03, and U01 mechanisms combined.

Figure S4

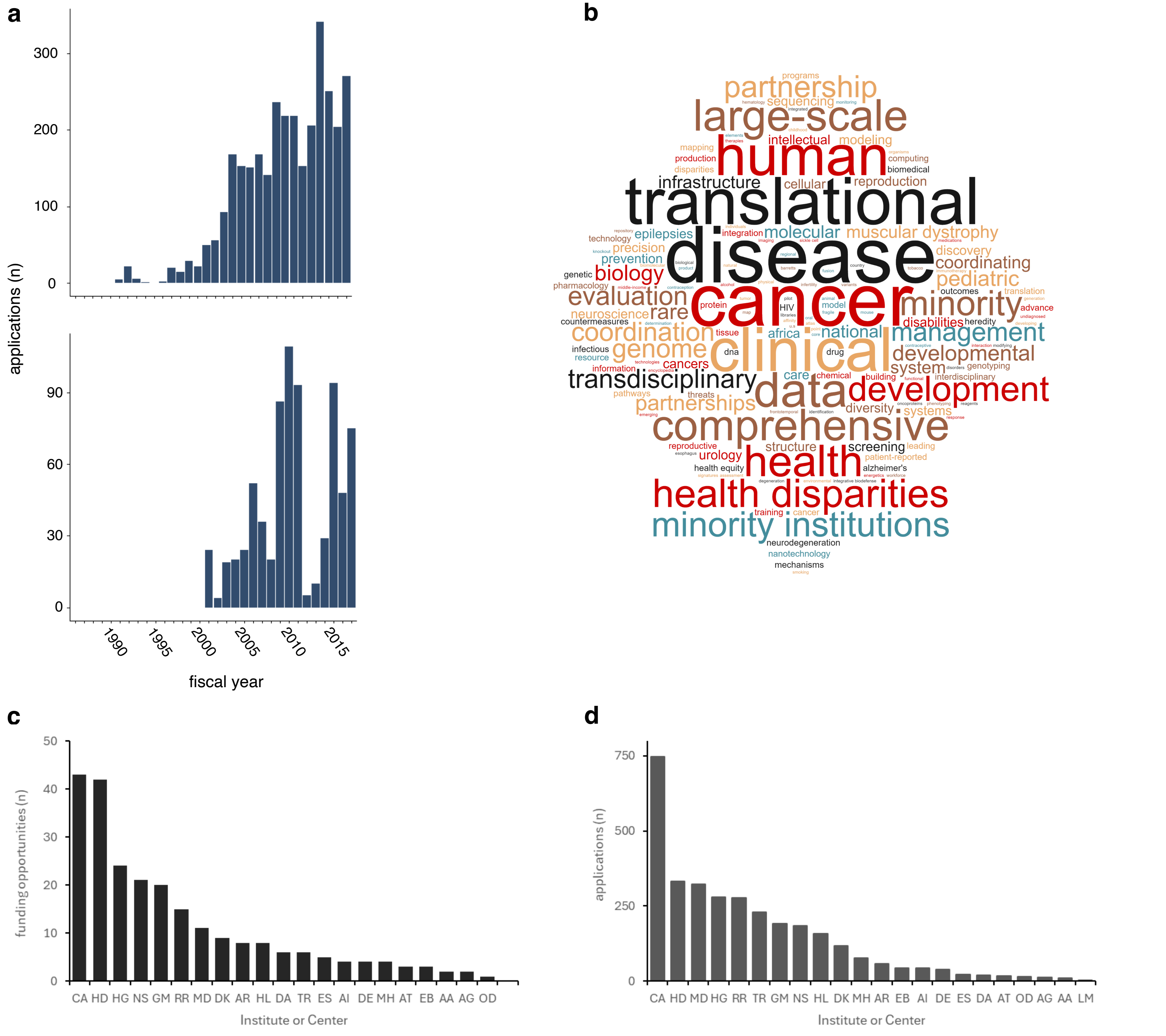

Figure S5. Analysis of U54 usage. (**a**) Number of U54 applications to NIH (top) or NCI (bottom) by year. (**b**) Themes of U54 Funding Opportunity Announcements. Word size correlates with frequency of appearance in the titles of U54 funding opportunity announcements, with larger words being more common and smaller words being less common. (**c**) Number of funding opportunities using the U54 across all years by Institute or Center (IC). (**d**) Number of U54 applications across all years, by IC.

Figure S5

Table S1. NIH-wide data on mechanism use.

Mechanism	First yr	Most yr	Last yr	First Count	Most Count	Last Count	Most:First	Last:First	Duration	active 2017	>30 appls	>6 yrs
R21	1986	2016	2017	4	16139	15257	4034.8	3814.3	31	1	1	1
R34	2003	2011	2017	1	717	585	717	585	14	1	1	1
U24	1992	2017	2017	1	301	301	301	301	25	1	1	1
R24	1989	2013	2017	1	586	209	586	209	28	1	1	1
U19	1993	2014	2017	1	272	142	272	142	24	1	1	1
P20	1986	2004	2017	2	352	163	176	81.5	31	1	1	1
UC4	2011	2017	2017	1	69	69	69	69	6	1	1	1
U54	1991	2014	2017	4	341	270	85.3	67.5	26	1	1	1
U44	1994	2010	2017	1	39	33	39	33	23	1	1	1
R25	1986	2011	2017	15	563	483	37.5	32.2	31	1	1	1
UM1	2011	2014	2017	2	157	48	78.5	24	6	1	1	1
R35	1986	2016	2017	51	958	861	18.8	16.9	31	1	1	1
R33	1999	2017	2017	19	318	318	16.7	16.7	18	1	1	1
DP3	2009	2012	2017	5	180	57	36	11.4	8	1	1	1
P41	1986	2010	2017	2	98	22	49	11	31	1	1	1
S06	1988	2006	2017	1	46	11	46	11	29	1	1	1
R44	1986	2016	2017	183	1905	1589	10.4	8.7	31	1	1	1
D43	1988	2011	2017	8	119	53	14.9	6.6	29	1	1	1
R42	1996	2016	2017	29	265	184	9.1	6.3	21	1	1	1
P42	1987	1995	2017	4	42	25	10.5	6.3	30	1	1	1
R15	1986	2017	2017	327	1812	1812	5.5	5.5	31	1	1	1
U01	1986	2015	2017	410	1964	1791	4.8	4.4	31	1	1	1
R03	1986	2004	2017	675	3796	2788	5.6	4.1	31	1	1	1
R41	1994	2016	2017	289	1511	1119	5.2	3.9	23	1	1	1
R56	2005	2008	2017	111	420	376	3.8	3.4	12	1	1	1
SC3	2007	2008	2017	25	103	79	4.1	3.2	10	1	1	1
P30	1986	2011	2017	36	220	108	6.1	3	31	1	1	1
G08	1986	1995	2017	22	210	56	9.5	2.5	31	1	1	1
R36	2004	2014	2017	16	52	39	3.3	2.4	13	1	1	1
UH2	2009	2014	2017	41	239	93	5.8	2.3	8	1	1	1
R43	1986	2004	2017	1676	5346	3719	3.2	2.2	31	1	1	1
U18	1990	2012	2017	5	49	10	9.8	2	27	1	1	1
R01	1986	2017	2017	18537	34205	34205	1.8	1.8	31	1	1	1
U34	2009	2015	2017	10	56	18	5.6	1.8	8	1	1	1
SC2	2007	2008	2017	47	130	76	2.8	1.6	10	1	1	1
P50	1986	2003	2017	100	261	156	2.6	1.6	31	1	1	1
P01	1986	2004	2017	336	571	311	1.7	0.9	31	1	1	1
S10	1991	2010	2017	406	1612	366	4	0.9	26	1	1	1
DP1	2004	2008	2017	239	515	208	2.2	0.9	13	1	1	1
SC1	2007	2008	2017	90	216	64	2.4	0.7	10	1	1	1
R18	1986	1991	2017	61	239	34	3.9	0.6	31	1	1	1
DP5	2011	2011	2017	108	108	44	1	0.4	6	1	1	1
U56	2001	2004	2017	8	45	2	5.6	0.3	16	1	1	1
DP2	2007	2007	2017	2181	2181	499	1	0.2	10	1	1	1
U43	1993	2012	2017	10	51	2	5.1	0.2	24	1	1	1
R37	1986	1987	2017	177	260	34	1.5	0.2	31	1	1	1
U10	1986	1994	2017	110	252	1	2.3	0	31	1	1	1
RF1	2012	2017	2017	7	134	134	19.1	19.1	5	1	1	0
UG1	2014	2016	2017	53	95	85	1.8	1.6	3	1	1	0
U2C	2014	2016	2017	1	42	14	42	14	3	1	1	0
UG3	2015	2017	2017	3	257	257	85.7	85.7	2	1	1	0
R50	2016	2016	2017	218	218	83	1	0.4	1	1	1	0
R61	2016	2017	2017	103	147	147	1.4	1.4	1	1	1	0
SB1	2016	2017	2017	72	104	104	1.4	1.4	1	1	1	0
OT2	2016	2017	2017	20	41	41	2.1	2.1	1	1	1	0
P60	1986	2012	2016	7	60	4	8.6	0.6	30	0	1	1
R55	1991	1991	2013	305	305	1	1	0	22	0	1	1

R29	1986	1995	2000	58	2516	1	43.4	0	14	0	1	1
S07	1991	1991	2003	628	628	117	1	0.2	12	0	1	1
RC1	2000	2008	2010	40	86	1	2.2	0	10	0	1	1
UC1	2000	2004	2005	8	130	93	16.3	11.6	5	0	1	0
R22	1986	1986	1989	96	96	64	1	0.7	3	0	1	0
DP7	2013	2013	2014	105	105	66	1	0.6	1	0	1	0
R23	1986	1986	1987	1075	1075	298	1	0.3	1	0	1	0
RL1	2007	2007	2007	51	51	51	1	1	0	0	1	0
PN1	2004	2004	2004	81	81	81	1	1	0	0	1	0
U42	1991	2006	2017	2	23	5	11.5	2.5	26	1	0	1
P40	1991	2003	2017	10	16	2	1.6	0.2	26	1	0	1
P51	1991	2008	2017	1	3	2	3	2	26	1	0	1
U41	1992	2012	2017	1	21	14	21	14	25	1	0	1
S21	2001	2011	2017	6	12	3	2	0.5	16	1	0	1
U2R	2003	2004	2017	1	24	11	24	11	14	1	0	1
R90	2004	2004	2017	17	17	4	1	0.2	13	1	0	1
UL1	2006	2007	2017	12	21	11	1.8	0.9	11	1	0	1
UH3	2013	2016	2017	1	18	12	18	12	4	1	0	0
UF1	2013	2016	2017	3	3	1	1	0.3	4	1	0	0
P2C	2014	2015	2017	6	26	6	4.3	1	3	1	0	0
UM2	2014	2017	2017	1	3	3	3	3	3	1	0	0
RM1	2015	2016	2017	7	17	5	2.4	0.7	2	1	0	0
OT3	2016	2017	2017	5	16	16	3.2	3.2	1	1	0	0
RC2	2017	2017	2017	11	11	11	1	1	0	1	0	0
G12	1991	2003	2014	8	9	7	1.1	0.9	23	0	0	1
M01	1991	1993	2008	19	24	7	1.3	0.4	17	0	0	1
S11	1994	2004	2011	1	14	2	14	2	17	0	0	1
UC7	2006	2016	2016	2	2	2	1	1	10	0	0	1
UH1	1996	2003	2004	5	5	3	1	0.6	8	0	0	1
RL5	2007	2014	2014	3	21	21	7	7	7	0	0	1
S22	2001	2001	2007	8	8	2	1	0.3	6	0	0	1
PN2	2005	2005	2010	20	20	6	1	0.3	5	0	0	0
UA5	2011	2011	2015	1	1	1	1	1	4	0	0	0
UG4	2016	2016	2016	11	11	11	1	1	0	0	0	0
PL1	2007	2007	2007	11	11	11	1	1	0	0	0	0
RC4	2010	2010	2010	6	6	6	1	1	0	0	0	0
RL9	2007	2007	2007	5	5	5	1	1	0	0	0	0
RC3	2010	2010	2010	2	2	2	1	1	0	0	0	0
R28	2013	2013	2013	1	1	1	1	1	0	0	0	0
RL2	2007	2007	2007	1	1	1	1	1	0	0	0	0

Table S2. Data on mechanism use at NCI.

Mechanism	First yr	Most yr	Last yr	First Count	Most Count	Last Count	Most:First	Last:First	Duration	active 2017	>30 appls	>6 yrs
R21	1986	2017	2017	4	13214	13214	3303.5	3303.5	31	1	1	1
R34	2003	2011	2017	1	717	585	717	585	14	1	1	1
R24	1989	2013	2017	1	585	209	585	209	28	1	1	1
R25	1987	2015	2017	1	496	422	496	422	30	1	1	1
U19	1993	2014	2017	1	271	141	271	141	24	1	1	1
U24	1992	2017	2017	1	217	217	217	217	25	1	1	1
P20	1989	2004	2017	2	352	79	176	39.5	28	1	1	1
R35	1988	2016	2017	6	777	716	129.5	119.3	29	1	1	1
U54	1991	2014	2017	4	312	195	78	48.8	26	1	1	1
UC4	2011	2017	2017	1	69	69	69	69	6	1	1	1
UM1	2011	2013	2017	2	105	47	52.5	23.5	6	1	1	1
P41	1986	2010	2017	2	98	22	49	11	31	1	1	1
S06	1988	2006	2017	1	46	11	46	11	29	1	1	1
DP3	2009	2012	2017	5	180	57	36	11.4	8	1	1	1
D43	1988	2011	2017	8	119	53	14.9	6.6	29	1	1	1
U10	1987	2011	2017	17	208	1	12.2	0.1	30	1	1	1
R44	1986	2016	2017	145	1556	1281	10.7	8.8	31	1	1	1
P42	1987	1995	2017	4	42	25	10.5	6.3	30	1	1	1
U18	1990	2012	2017	5	49	10	9.8	2	27	1	1	1
G08	1986	1995	2017	22	210	56	9.5	2.5	31	1	1	1
R33	2001	2006	2017	11	105	10	9.5	0.9	16	1	1	1
P30	1986	2011	2017	22	205	98	9.3	4.5	31	1	1	1
R42	1996	2016	2017	26	221	151	8.5	5.8	21	1	1	1
R18	1986	1991	2017	35	236	34	6.7	1	31	1	1	1
R03	1986	2004	2017	513	3428	1973	6.7	3.8	31	1	1	1
U44	2002	2010	2017	6	39	33	6.5	5.5	15	1	1	1
R15	1986	2015	2017	279	1576	1527	5.6	5.5	31	1	1	1
U34	2009	2015	2017	10	56	18	5.6	1.8	8	1	1	1
R41	1994	2016	2017	235	1191	865	5.1	3.7	23	1	1	1
U01	1986	2015	2017	319	1408	1070	4.4	3.4	31	1	1	1
SC3	2007	2008	2017	25	103	79	4.1	3.2	10	1	1	1
UH2	2009	2015	2017	38	154	75	4.1	2	8	1	1	1
S10	1991	2010	2017	406	1612	366	4	0.9	26	1	1	1
R56	2005	2008	2017	110	384	370	3.5	3.4	12	1	1	1
R43	1986	2004	2017	1267	4291	3022	3.4	2.4	31	1	1	1
R36	2004	2014	2017	16	52	39	3.3	2.4	13	1	1	1
SC2	2007	2008	2017	47	124	76	2.6	1.6	10	1	1	1
SC1	2007	2008	2017	89	210	59	2.4	0.7	10	1	1	1
P50	1986	1999	2017	99	225	109	2.3	1.1	31	1	1	1
DP1	2004	2008	2017	239	515	206	2.2	0.9	13	1	1	1
R01	1986	2017	2017	15872	28145	28145	1.8	1.8	31	1	1	1
P01	1986	2004	2017	263	440	216	1.7	0.8	31	1	1	1
R37	1986	1987	2017	150	223	34	1.5	0.2	31	1	1	1
DP5	2011	2011	2017	108	108	44	1	0.4	6	1	1	1
DP2	2007	2007	2017	2181	2181	493	1	0.2	10	1	1	1
UG3	2015	2017	2017	3	245	245	81.7	81.7	2	1	1	0
RF1	2012	2017	2017	7	134	134	19.1	19.1	5	1	1	0
OT2	2016	2017	2017	20	41	41	2.1	2.1	1	1	1	0
U2C	2015	2016	2017	23	42	14	1.8	0.6	2	1	1	0
UG1	2015	2016	2017	54	95	85	1.8	1.6	2	1	1	0
SB1	2016	2017	2017	72	104	104	1.4	1.4	1	1	1	0
R61	2016	2017	2017	103	147	147	1.4	1.4	1	1	1	0
R29	1986	1995	2000	47	2172	1	46.2	0	14	0	1	1
P60	1986	2012	2016	7	60	4	8.6	0.6	30	0	1	1
RC1	2000	2008	2010	40	86	1	2.2	0	10	0	1	1
R55	1991	1991	2013	250	250	1	1	0	22	0	1	1
S07	1991	1991	2003	628	628	117	1	0.2	12	0	1	1

UC1	2000	2004	2005	8	130	93	16.3	11.6	5	0	1	0
DP7	2013	2013	2014	105	105	66	1	0.6	1	0	1	0
RL1	2007	2007	2007	47	47	47	1	1	0	0	1	0
PN1	2004	2004	2004	81	81	81	1	1	0	0	1	0
R22	1986	1986	1989	96	96	64	1	0.7	3	0	1	0
R23	1986	1986	1987	940	940	265	1	0.3	1	0	1	0
U2R	2003	2004	2017	1	24	11	24	11	14	1	0	1
U41	1992	2012	2017	1	21	14	21	14	25	1	0	1
U42	1991	2006	2017	2	23	5	11.5	2.5	26	1	0	1
P51	1991	2008	2017	1	3	2	3	2	26	1	0	1
S21	2001	2011	2017	6	12	3	2	0.5	16	1	0	1
UL1	2006	2007	2017	12	21	11	1.8	0.9	11	1	0	1
P40	1991	2003	2017	10	16	2	1.6	0.2	26	1	0	1
U56	2003	2003	2017	12	12	2	1	0.2	14	1	0	1
R90	2004	2004	2017	17	17	4	1	0.2	13	1	0	1
UH3	2013	2016	2017	1	15	9	15	9	4	1	0	0
P2C	2014	2015	2017	6	26	6	4.3	1	3	1	0	0
OT3	2016	2017	2017	5	16	16	3.2	3.2	1	1	0	0
UM2	2014	2017	2017	1	3	3	3	3	3	1	0	0
RM1	2015	2016	2017	7	17	5	2.4	0.7	2	1	0	0
UF1	2013	2016	2017	3	3	1	1	0.3	4	1	0	0
RC2	2017	2017	2017	11	11	11	1	1	0	1	0	0
U43	2017	2017	2017	1	1	1	1	1	0	1	0	0
S11	1994	2004	2011	1	14	2	14	2	17	0	0	1
RL5	2007	2014	2014	2	21	21	10.5	10.5	7	0	0	1
M01	1991	1993	2008	19	24	7	1.3	0.4	17	0	0	1
G12	1991	2003	2014	8	9	7	1.1	0.9	23	0	0	1
UC7	2006	2016	2016	2	2	2	1	1	10	0	0	1
UH1	1996	2003	2004	5	5	3	1	0.6	8	0	0	1
S22	2001	2001	2007	8	8	2	1	0.3	6	0	0	1
PN2	2005	2005	2010	20	20	6	1	0.3	5	0	0	0
UG4	2016	2016	2016	11	11	11	1	1	0	0	0	0
UA5	2015	2015	2015	1	1	1	1	1	0	0	0	0
R28	2013	2013	2013	1	1	1	1	1	0	0	0	0
RC4	2010	2010	2010	6	6	6	1	1	0	0	0	0
RC3	2010	2010	2010	2	2	2	1	1	0	0	0	0
PL1	2007	2007	2007	10	10	10	1	1	0	0	0	0
RL9	2007	2007	2007	4	4	4	1	1	0	0	0	0
RL2	2007	2007	2007	1	1	1	1	1	0	0	0	0
